# Prosocial Behavior Can Moderate the Relationship Between Rumination and Mindfulness

**DOI:** 10.3389/fpsyt.2020.00289

**Published:** 2020-04-17

**Authors:** Yao Meng, Gang Meng

**Affiliations:** ^1^ Department of Psychology, Nanjing University, Nanjing, China; ^2^ School-Based Mental Health Center, Zibo Vocational Institute, Zibo, China

**Keywords:** rumination, mindfulness, prosocial behavior, moderating effect, mental health

## Abstract

**Objective:**

Rumination, which is a coping style to distress, has become a common mode of thinking about mental illnesses such as depression and anxiety. Improving mindfulness is an effective way to help people cope with rumination. Individuals who had higher prosocial behaviors reported a high level of mindfulness. This study aimed to explore whether prosocial behavior helps individuals with high-level rumination improve their mindfulness, and explain the reason why prosocial behavior can influence the relationship between mindfulness and rumination.

**Methods:**

Introducing prosocial behavior situations, the first study chose 51 high-level rumination and 53 low-level rumination participants and measured the influence of prosocial behavior on mindful attention awareness in the present moment. In the second study, a questionnaire was conducted among 261 participants to explore the moderating effect of prosocial behavior between rumination and mindfulness.

**Results:**

In individuals with high-level rumination, ΔMAAS (mindful attention awareness scale) (posttest-baseline) scores in the prosocial behavior condition were significantly higher compared to those in the control condition (p=0.003). Meanwhile, prosocial behavior played a moderating effect between reflective pondering of rumination and mindfulness (R^2^^=^ 0.03, p=0.004).

**Conclusions:**

Encouraging prosocial behavior is an effective way to improve mindfulness in highly ruminative individuals.

## Introduction

Rumination is a coping style to distress that involves a repetitive and passive focus on symptoms of distress and the likely causes and consequences of these symptoms (1). Studies have shown that rumination is highly correlated with negative emotion; people have symptoms such as depression, anxiety, fear, and addiction largely because their attention is focused on deep rumination from which they find it difficult to extricate themselves (2–6). Therefore, an important aim in mental health or psychotherapy is to find ways to cope with rumination.

Initial research has suggested that distraction is a coping skill that decreases rumination by discarding unpleasant thoughts and feelings, often by focusing on other thoughts or activities (7). However, this strategy only works for a short time and under certain conditions. Mindfulness appears to be a different construct than distraction and may have greater potential as an alternative to ruminative thought processes (8, 9). Mindfulness refers to the self-regulation of attention in the present moment without analyzing and criticizing, which involves two components. First, mindfulness involves bringing one's full attention to the present moment, rather than dwelling on past events or future possibilities (10). Second, mindfulness is characterized by an attitude of nonjudgmental acceptance of internal and external events (8). Several studies have indicated that heightened mindfulness can decrease rumination, which in turn results in less depression and anxiety (11–14). Shapiro trained 83 college students in mindfulness-based stress reduction (MBSR) to reduce successfully the degree of rumination (15). While monitoring the 42-day mindfulness-based intervention (MBI) process of 45 participants, Andreotti found that the degree of rumination decreased significantly after 1 week and 1 month (16). Mindfulness cultivates nonjudgmental, nonreactive attention to the present moment; however, it can also be experienced in other ways, except for professional mindfulness training (17, 18). Prosocial behaviors include a range of positive and friendly behaviors that individuals display toward others (19). According to previous studies, individuals who exhibited a higher prosocial behavior trait reported a high level of mindfulness, especially the mindful attention awareness (20, 21). The reason why prosocial behaviors relate to mindfulness may be based on the relationships of their features. On the one hand, the premise of prosocial behavior is being aware of the needs of others in the present; this present-focused attention increases positive emotions and mindful awareness of individuals (19, 22). On the other hand, within helping situations, it is the nonjudgmental acceptance attitude that may allow people to disengage from their own emotions and focus on those in need of help (23). In particular, individuals with a heightened tendency to ruminate may likely improve their mindful awareness in case they are able to invest cognitive resources on the present moment, without assigning a judgment value to the emotions experienced (24). That is to say, prosocial behavior can help individuals with high-level rumination to improve their mindful awareness. Therefore, introducing the prosocial behavior task, the first study aimed to explore to what extent the prosocial behavior helps individuals with high-level rumination improve their mindful awareness.

It is acknowledged that there are negative correlation between rumination and mindfulness (3, 25). If the first study indicated that engaging in prosocial behavior helps individuals with a high level of rumination improve their mindful perception, this would imply that prosocial behavior may moderate the relationship between rumination and mindful awareness. What is the reason for this? In fact, Treynor et al. (26) found two distinct components of rumination emerging from the following items: *reflective pondering,* a “purposeful turning inward to engage in cognitive problem-solving to alleviate one's depression,” and *brooding*, “a passive comparison of one's current situation with some unachieved standard” (26). To some extent, both of these components can be seen as a repetitive thinking style, and even when it is problem-orientated, it may not always be the most adaptive approach to experience mindfulness and positive emotion (1, 27). Furthermore, although both of them showed an association with more depression concurrently, compared to the brooding component, the reflective pondering component is a more adaptive problem-orientated ability for humans (26, 28). Research showed that the ability to be mindful may represent an important prerequisite for adaptive engagement in reflective pondering (12). Thus, it is likely that problem-orientated reflective pondering helps individuals engage in the helping situation and improve mindfulness perception. As a result, using the ruminative response scale (RRS), consisting of reflective pondering and brooding components, the second study assessed the reason why prosocial behavior is a potential moderator of the relationship between mindfulness and rumination. One goal of the current study was, therefore, to test whether the level of prosocial behavior moderates the relationship between reflective pondering and mindfulness.

Our first study measured whether prosocial behavior could help individuals with high-level rumination improve their mindful awareness. Because the experiment was introduced in scenes requiring participants to imagine engaging in prosocial behavior, the mindful attention awareness (measured by mindful attention awareness scale, MAAS), which represents a mindful awareness variable in the present moment (29, 30), was used to measure the change of mindful states over time. Our first hypothesis was that for individuals with high-level rumination, their mindful attention awareness of the experimental group after intervention would be significantly higher than that of the control group. Using a questionnaire analysis, the second study measured the reason why prosocial behavior was a potential moderator of the relationship between mindfulness and rumination. According to the reviews, among all components of rumination, the reflective pondering of rumination helps individuals engage in helping situation and improve mindfulness perception. Our second hypothesis was that prosocial behavior can moderate the relationship between reflective pondering and mindfulness. Besides, previous study reported that MAAS just focused on the presence or the absence of attention to and the awareness of what was occurring in the present rather than other facts of mindfulness such as acceptance and observing (30). Therefore, in the second study, the Five Facet Mindfulness Questionnaire (FFMQ), comprising reasonable psychometric properties, is currently the one that assesses a more comprehensive concept of mindfulness (31).

## Study 1

### Methods

#### Participants

Two hundred participants were recruited by posting flyers at local university buildings and through an advertisement on a website. Upon initial contact, all participants were asked to fill in the Ruminative Responses Scale (RRS) and participate in an online interview, which was conducted to establish eligibility for the study through an assessment of mental and physical health history. Exclusionary criteria for participants included a history of psychiatric disorders and the current use of psychotropic drugs or alcohol and other drug abuse. Three participants were excluded for their history of a psychiatric disorder, two participants were excluded for taking psychotropic drugs, and seven participants were excluded because they did not fill out all the questions nor had only one option for all questions in the RRS. The resulting dataset included 188 students (88 male, 100 female). According to the RRS score of each participant, out of the total of 188 students, 50 participants (27%) were in the high-level rumination group, and 50 participants (27%) were in the low-level rumination group. According to the random matching condition, half of each group was placed in the experimental condition while the remaining half was put in the control condition. However, an additional eight participants were excluded because they did not write down what they thought under different conditions. In order to make sure there were more than 20 people in each condition, we added three participants in each of them. This implied the study actually used top and bottom 30% as the criterion of higher and lower rumination. The RRS scores of the higher and lower rumination were significantly different (p=0.003). Ultimately, the data of the high-RRS group (23 participants in the experimental condition and 28 participants in the control condition) and the low-RRS group (28 participants in the experimental condition and 25 participants in the control condition) were included in the statistical analysis.

The study was approved by the local ethics committee and carried out in accordance with the Declaration of Helsinki. All participants signed informed consent forms.

#### Procedure

Participants in two groups were invited to a quiet lecture theatre at the same time. First, after 10 min of rest, everyone filled out the MAAS. Then, those in the experimental condition were asked to consider some specific ways to help others in created helping situations while the remaining half in the control condition were primed with irrelevant stimulation. This process took 15 min. After these, the MAAS was filled again. Finally, all subjects were paid for their participation.

#### Materials

The RRS was compiled by Nolen-Hoesksema (7), and the Chinese version was revised by Xiu and Hong fei (32). It consists of 22 items and is divided into three subscales related to depressive symptoms (12 items; e.g., “Think about how alone you feel”), the Brooding scale (5 items; e.g., “Think why do I always react this way?”), and the Reflection scale (5 items; e.g., “Go away by yourself, and think about why you feel this way”). It was scored using a Likert-type scale of 4 points ranging from 1 (almost never) to 5 (almost always). The results of the RRS measurement for Chinese college students suggest that the Cronbach's coefficient is 0.86 (33). In this study, the alpha coefficient of the questionnaire was 0.79.

The MAAS was compiled by Brown and Ryan and assesses mindful awareness of individuals (30). It focuses on the presence or absence of attention and awareness of what is occurring in the present (34, 35), which can assess individual differences in the frequency of mindful states over time (30). The MAAS has 15 items such as “I find myself preoccupied with the future or the past.” It was scored using a six-point Likert-type scale ranging from 1 (*almost always*) to 6 (*almost never*). Higher scores reflect higher mindful attention awareness. The Cronbach's coefficient of the Chinese version is 0.89 (36). In this study, the alpha coefficient of the questionnaire was 0.87.

The task for those in the experimental condition was composed of prosocial behavior response missions, which were developed by Chinese scholar Ma based on characteristics of Chinese prosocial behavior (37). Informed by situations of social need in China, this task included six situations that could arouse the participants' desire to help others successfully (e.g., “Zhou, a freshman of your major, just came to the city from a remote village in Yunnan province for study. He has difficult family economic conditions, and his Mandarin Chinese is not good enough to communicate with others. You are his classmate; what should you do to help him?”). For each story situation, participants were asked to imagine and concentrate on the situation, and then write down two or more ways to help. Further, the instructions asked the participants to delineate the steps necessary to those methods of helping. These response missions, printed individually on note cards, were designed to influence the thought content of the participants by requiring them to focus their attention and imagine how they could help. The aim is to take the attention of the participants away from themselves and shift it to help others. Ma asserted these missions could motivate prosocial behaviors because people showed a higher proportion of donations after similar situations were in the news (37).

The tasks for those in the control condition were distraction missions created by Morrow and Nolen-Hoekseina (35). Participants engaged in the distraction missions were asked to focus on items not related to themselves and related to imagining external events, similar to the experimental group; however, these were not centered on helping others (e.g., “if a ship is heading for the coast, please describe the scene you imagined” and “the layout of a typical classroom, please describe the scene you imagined”). The distraction missions have been previously rated as equally neutral by nondysphoric judges (38). Consistent with the tasks of those in the experiment condition, participants were asked to read six questions printed individually on note cards and answer them in turn.

### Study Design and Statistical Analysis

To avoid the effect of repeated measurements, we adopted 2 (condition: prosocial behavior and control) × 2 (group: high-level rumination and low-level rumination) between-subject design. With these types of designs, the number of people assigned to each condition was random, causal estimates are obtained by comparing the change of MAAS in experimental condition with the change of those in control condition. Given that the baseline data of MAAS significantly differed in the high and low rumination groups, we used the D-value (ΔMAAS= posttest-baseline) as the dependent variable to indicate the effect sizes. Meanwhile, 2 × 2 intergroup ANOVAs were performed (p < 0.05). All data were analyzed using SPSS 22.0.

### Results

#### Demographic Variables and Correlations

According to their scores on the RRS, we divided the participants into a highly ruminative group and a lowly ruminative group. The result of an independent sample *T*-test displayed that the differences of RRS [t(102)=21.33,p < 0.001] and MAAS [t(102) =−13.85, p < 0.001] between the two groups were significant.

Furthermore, in the highly ruminative group, there were no differences in age, RRS and MAAS [all t(49) < 1.87, all p > 0.09] between those in the experimental and control conditions. Likewise, in the lowly ruminative group, there were no differences in age, RRS and MAAS [all t(51) < 0.18, all p > 0.38] between the experimental and control conditions. **Table 1** shows the mean and standard deviation of age and questionnaires.

**Table 1:** Demographic variables and questionnaire scores.

	High Rumination		Low Rumination	
	Prosocial condition	Control condition	Prosocial condition	Control condition
Age	18.30 ± 2.15	18.17 ± 1.44	18.24 ± 2.12	18.36 ± 1.82
RRS	54.65 ± 6.20	53.75 ± 6.87	32.68 ± 3.58	32.84 ± 2.81
MAAS	51.34 ± 5.37	48.23 ± 6.02	66.32 ± 6.11	66.64 ± 6.92

RRS, ruminative responses scale; MAAS, mindful attention awareness scale.

#### Mindfulness Measurement Under Experimental and Control Condition

The results revealed that the main effect of the experimental group (F_(1, 102)_=15.21, p < 0.001) and the control (F_(1, 102)_=9.04, p < 0.001) were significant. The ΔMAAS (posttest-baseline) of those in the experimental condition was higher than those in the control condition. In addition, significant differences also existed in interaction of group and conditions (F_(1, 102)_=4.12, p=0.045). Simple effect analysis indicated that the ΔMAAS of the highly ruminative group was significantly higher than that of the lowly ruminative group in the prosocial behavior condition (F_(1, 102)_=14.84, p < 0.001); further, for the highly ruminative group, the ΔMAAS in the prosocial behavior condition was significantly higher than that in the control condition (F_(1, 102)_=9.55, p=0.003). **Table 2** shows the mean and standard deviation of MAAS in different stages.

**Table 2:** The MAAS score in difference stages.

			MAAS			N
			Baseline	Post test	Posttest-baseline (Δ)	
HR	Prosocial condition		51.34 ± 5.37	56.78 ± 6.33	**4.83 ± 3.52** ^a^ ^,^ ^b^	23
	Control condition		48.23 ± 6.02	50.32 ± 5.40	1.63 ± 2.32	28
LR	Prosocial condition		66.32 ± 6.11	67.32 ± 5.77	1.07 ± 1.56	28
	Control condition		66.64 ± 6.92	67.08 ± 6.85	0.40 ± 2.45	25

HR, high rumination; LR, low rumination; MAAS, mindful attention awareness scale. Baseline, the data of MAAS measured before test; Post test, the data of MAAS measured after test. Posttest-baseline, the data of post test minus the data of baseline.

a Significantly different from the prosocial condition in LR group.

b Significantly different from the control condition in HR group.

## Study 2

### Method

#### Participants

The second study conducted was a questionnaire survey, including the RRS, Five Facet Mindfulness Questionnaire (FFMQ), and the Prosocial Tendency Measurement (PTM), on 300 college students. These participants were recruited by posting flyers at a local university and through an advertisement on a website. None were psychology majors or had any experience of social work. Thirty-four participants were excluded because they did not fill out all the questions, and five participants were excluded for their history of psychiatric disorders, leaving a total of 261 participants (144 male and 177 female). There were no significant differences in age between males and females (p=0.31). **Table 3** is the sociodemographic profile of the participants.

**Table 3:** Sociodemographic profile of the participants (N=261).

	n	%	M	SD
**Age**	261		20.68	2.4
Male	144	55.2%	20.55	2.1
Female	117	45.8%	21.04	2.8
**Education level**				
Undergraduate	222	85.1%	–	–
Post graduate	39	14.9%	–	–
**Parental education**				
UNIV DIP or above	88	33.7%	–	–
High school	132	50.6%	–	–
Junior high school	41	15.7%	–	–

UNIV DIP, university diploma.

#### Materials

The FFMQ is a 39-item questionnaire that measures five facets of mindfulness (31): observing (eight items; e.g., “I notice the smells and aromas of things”), describing (eight items; e.g., “I'm good at finding the words to describe my feelings”), acting with mindful awareness (eight items; e.g., “I am easily distracted”), nonjudging (eight items; e.g., “I criticize myself for having irrational or inappropriate emotions”), and nonreactivity (seven items; e.g., “I watch my feelings without getting lost in them”). Items were scored on a five-point Likert-type scale ranging from 1 (never or very rarely true) to 5 (very often or always true). Higher scores indicate more mindfulness. The Cronbach's coefficient of the Chinese version is 0.81 (39). In this study, the alpha coefficient of the questionnaire was 0.85.

PTM was developed by Carlo and Randall measuring trait prosocial behavior tendencies on six subscales (40): altruism (five items; e.g., “When people ask me to help them, I don't hesitate”), anonymous prosocial behavior (five items; e.g., “I prefer to donate anonymously”), compliant prosocial behavior (two items; e.g., “I feel that if I help someone, they should help me in the future”), dire prosocial behavior (three items; e.g., “It is easy for me to help others when they are in a dire situation”), emotional prosocial behavior (four items; e.g., “I respond to helping others best when the situation is highly emotional”), and public prosocial behavior (four items; e.g., “I will try my best to help others in the public”). It was scored using a Likert-type scale of five points ranging from 1 (completely out of line) to 5 (completely suitable). The higher the score, the more prosocial tendency the individual has. The Chinese version of the scale was revised with a college student cohort, and its Cronbach's coefficient is 0.85 (41). In this study, the alpha coefficient of the questionnaire was 0.79.

The RRS was the same as in Study 1.

### Statistical Analysis

The data were analyzed using SPSS 22.0. The premise of moderation analysis is that a correlation between independent and dependent variables exists, and the moderator variable is a reliable predictor of the dependent variable. Thus, Pearson's correlations were calculated between different subscales of RRS, FFMQ, and PTM scores. Second, the moderation analysis was conducted using the Hayes PROCESS macro in SPSS (42). In order to do that, independent and moderator variables needed to be centralized. We used 5,000 bootstrap samples, and biases were corrected at 95% confidence intervals (CIs) to calculate the indirect effect of each variable. The presence of a significant effect is denoted if zero is not included by the upper and lower bound of 95% CI.

### Results

#### Demographic Variables and Correlations

Initially, we transformed all RRS, FFMQ, and PTM scores into standard Z scores. We compared the correlations among every subscale of RRS, PTM and FFMQ. The results indicated that every subscale of RRS (all p < 0.05) correlated significantly with FFMQ, and that there was a significant correlation between PTM and FFMQ (p < 0.001). Although the correlation between the reflective pondering of RRS and PTM was not significant (p=0.75), PTM could be used as a moderator variable for analysis. Furthermore, no gender differences were found in the RRS, FFMQ, and PTM (all p > 0.205). The correlations are presented in **Table 4**.

**Table 4:** The descriptive statistics for Z scores of questionnaires and their correlation.

	M	SD	1	2	3	4	5
1 RRS	2.04	0.59	–	–	–	–	–
2 RRS-Depression	2.01	0.81	0.909**	–	–	–	–
3 RRS-Brooding	2.05	0.76	0.862**	0.837**	–	–	–
4 RRS-Reflection	2.06	0.69	0.594**	0.262**	0.149**	–	–
5 FFMQ	3.13	0.29	−0.399**	−0.390**	−0.375**	−0.158*	–
6 PTM	3.33	0.41	−0.113	−0.146**	−0.128*	0.020	0.297**

**p < 0.001, *p < 0.05.. RRS, ruminative responses scale; FFMQ, Five Facet Mindfulness Questionnaire; PTM, prosocial tendency measurement.

#### Moderation Analysis

**Figure 1:**
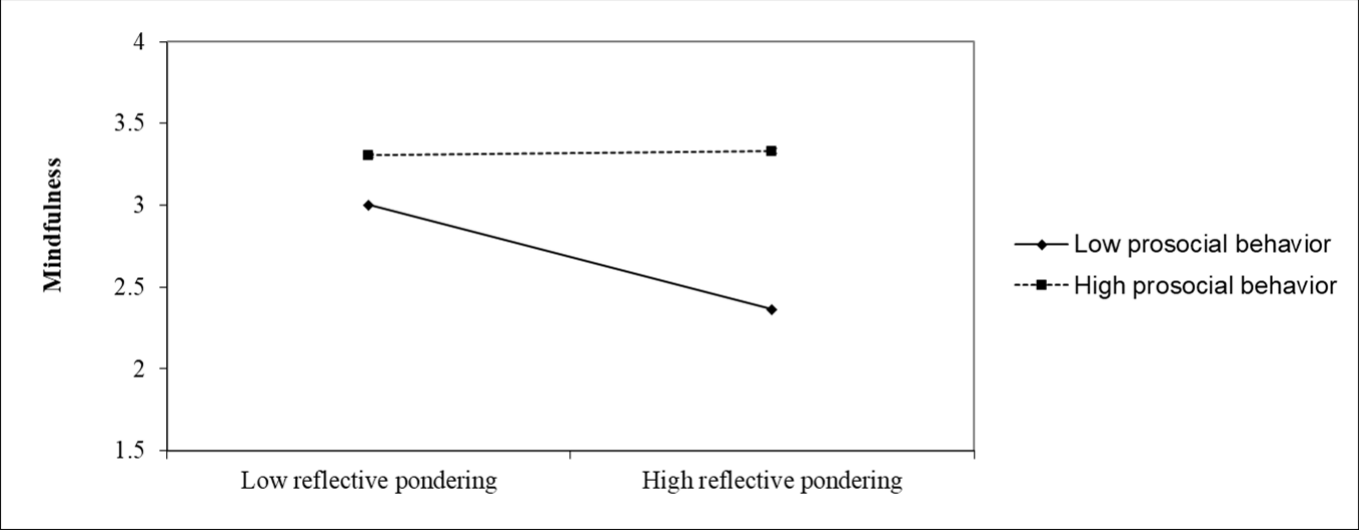
The predictive effect of prosocial behavior.

The outcomes of the interaction tests for potential moderators are shown in **Table 5**. A significant moderation effect existed in prosocial behavior on the relationship between the reflective pondering component of rumination and mindfulness (R^2^ = 0.03, F=8.27, p = 0.004). In order to clearly reveal the direction of the moderator, we divided the high- prosocial behavior group (mean value + 1 standard deviation) from the low- prosocial behavior group (mean minus value −1 standard deviation) so that the predictive effect of reflective pondering on mindfulness could be clearly shown under the different levels of prosocial behavior. For individuals who demonstrate highly reflective pondering, the higher prosocial behavior trait they have, the more experience of mindfulness. **Figure 1** shows the predictive effect of the moderator.

**Table 5:** Moderating effects of prosocial behavior on the relationship between rumination and mindfulness (N=261).

	Variables	β	SE	*P* value	LLCI	ULCI
RRS-1	Constant	−.003	.058	.955	−.117	.110
	PTM	.318	.058	< 0.001	.204	.432
	**RRS-Reflection**	−.153	.058	.009	−.268	-.038
	**Interaction**	.166	.057	.004	.052	.279
RRS-2	Constant	.015	.056	.795	−.095	.124
	PTM	.260	.056	< 0.001	.149	.371
	**RRS-Depression**	−.353	.056	< 0.001	−.463	−.243
	**Interaction**	.098	.059	.092	−.016	.216
RRS-3	Constant	.013	.056	.821	−.097	.122
	PTM	.268	.056	< 0.001	.156	.378
	**RRS-Brooding**	−.345	.056	< 0.001	−.455	−.235
	**Interaction**	.099	.059	.093	−.017	.215

RRS, ruminative responses scale; RRS-1 is reflection subscale, RRS-2 is depressive subscale, and RRS-3 is brooding subscale; PTM, prosocial tendency measurement. The interaction term was generated by multiplying the mean-centered values of PTM and every subscale of RRS. β, unstandardized coefficient; SE, standard error; CI, confidence interval (LLCI lower bound CI; ULCI upper bound CI).

## Discussion

Our study found that engaging in prosocial behavior is a way to help highly ruminative individuals improve their mindfulness, and prosocial behavior can moderate the relationship between reflective pondering component of rumination and mindfulness. This implied that reflective pondering of rumination helps individuals engage in a helping situation and improves mindfulness perception.

The first study investigated to what extent prosocial behavior helps individuals with high-level rumination improve their mindful awareness when they are immersed in the helping scene. The results, consistent with our first hypothesis, showed that for the individuals with high-level rumination, the mindful awareness of the experimental condition was significantly higher than that of the control condition. Compared with the distraction task, which emphasized focusing on other thoughts or activities, the prosocial behavior task asked the participants to focus on the present and the awareness of the needs of others. For one thing, this present-focused attention aroused people's empathy and psychological flexibility, which allowed people to disengage from their own emotions and obtain more mindful attention awareness (43–45). For another, it is the nonjudgmental acceptance attitude that may allow people to disengage from their own emotions and focus on those in need of help (23). For this reason, the participants were asked to perform helping behaviors based on the specific needs of others; this action implied that they must reduce their internal or external judgments and emotion, which to some extent increased their level of mindful awareness. Furthermore, individuals with a heightened tendency of rumination may likely improve their mindful awareness and invest cognitive resources on the present moment, without assigning a judgment value to the emotions experienced (24). This may explain why individuals with high-level rumination can improve their mindful awareness through prosocial problem-solving. However, our results found that for individuals with low-level rumination, the difference between the two conditions is not significant. Researchers reported that individuals with low-level rumination and high-level mindfulness exhibit stronger flexibility and efficiency in switching attention (46, 47). A 15-min experience of prosocial behavior may be common to them and not sufficient to cause significant changes in mindfulness levels compared to distraction condition. Individuals with high-level rumination did not have much voluntary empathy experience (27), and passively shifting their attention from themselves to others may be an effective way for them to increase mindful awareness. Above all, the occurrence of prosocial behavior can increase the mindful awareness of individuals with high-level rumination.

The first study demonstrated that individuals with high-level rumination can improve their mindful awareness through prosocial problem-solving, which implied that prosocial behavior could moderate the relationship between rumination and mindfulness. The second study aimed to identify the reason for this. Previous studies indicated that compared to the depressive and brooding components of rumination, the reflective pondering component is a more adaptive problem-orientated ability for humans. The ability to be mindful may represent an important prerequisite for adaptive engagement in reflective pondering of rumination (12, 26). Consistent with our hypothesis, the results revealed that prosocial behavior can moderate the relationship between reflective pondering and mindfulness. For highly reflective pondering individuals, the higher the prosocial behavior trait is, the more experience of mindfulness. Unlike other components of rumination, the reflection is a more adaptive strategy per se. Ramel proposed that the ability to be mindful represent an important prerequisite for adaptive engagement in reflective pondering (12). However, the effects of reflection were not consistently adaptive but differed, depending on the type of coping styles; for example, in those with a less active coping style, reflection was related to elevated levels of depression and decreased mindfulness, whereas in those with a more active coping style, this was not the case (48). In other words, the prosocial behavior may be an active style of coping for ruminators. Prosocial behavior can enhance the psychological flexibility of individuals to a certain extent and enable individuals to release their negative emotion. It is a positive active trait for people (49, 50). In short, reflective pondering helps individuals engage in a helping situation and improve mindfulness perception. The prosocial behavior moderates the relationship between reflective pondering and mindfulness.

To sum up, our study found that engaging in prosocial behavior is a way to help highly ruminative individuals improve their mindfulness, and prosocial behavior can moderate the relationship between reflective pondering component of rumination and mindfulness. This implied that reflective pondering of rumination helps individuals engage in a helping situation and improves mindfulness perception. However, this study has certain limitations. First, the assessment of mindful awareness is based on self-report measures; therefore, there is the possibility of a response bias. More measurements (e.g., cognition task and physical index) need to be added in future studies. Second, the imagination of prosocial behavior is different from engagement in real situations. Thus, effective simulations of prosocial behavior need to be developed for academic research.

## Data Availability Statement

The data cannot be shared at this time as the data also forms part of an ongoing study. After all the researches are done, the data will be made available by the authors. Requests to access these datasets should be directed to the corresponding author.

## Ethics Statement

The studies involving human participants were reviewed and approved by ethical standards of the research committee of Nanjing University. The patients/participants provided their written informed consent to participate in this study.

## Author Contributions

All authors contributed to the study conception and design. Material preparation, data collection were performed by GM. The data were analyzed and first draft of the manuscript was written by YM. All authors commented on previous versions of the manuscript and read and approved the final manuscript.

## Conflict of Interest

The authors declare that the research was conducted in the absence of any commercial or financial relationships that could be construed as a potential conflict of interest.
